# Further development of silk sericin as a biomaterial: comparative investigation of the procedures for its isolation from *Bombyx mori* silk cocoons

**DOI:** 10.1007/s40204-016-0052-8

**Published:** 2016-07-19

**Authors:** Traian V. Chirila, Shuko Suzuki, Natalie C. McKirdy

**Affiliations:** 1 Queensland Eye Institute, South Brisbane, QLD 4101 Australia; 2 Science & Engineering Faculty, Queensland University of Technology (QUT), Brisbane, QLD 4001 Australia; 3 Faculty of Medicine & Biomedical Sciences, The University of Queensland (UQ), Herston, QLD 4029 Australia; 4 Australian Institute of Bioengineering & Nanotechnology (AIBN), The University of Queensland (UQ), St Lucia, QLD 4072 Australia; 5 Faculty of Science, The University of Western Australia (UWA), Crawley, WA 6009 Australia

**Keywords:** *Bombyx mori* silk, Silk proteins, Sericin, Methods of extraction, Molecular mass distribution, Electrophoresis, FTIR analysis

## Abstract

There is significant research dedicated to fibroin and sericin, the two major proteinaceous components of the silk threads produced by the domesticated silkworm, *Bombyx mori*. While fibroin is accepted as an established biomaterial, sericin (BMSS) has been largely neglected in this respect on the account of a hypothetical allergenic activity. Research over the past decade, including our previous study (Prog Biomater 2:14, 2013), demonstrated the biocompatibility of sericin and feasibility of its use as a biomaterial. However, the current procedures for isolating BMSS from the raw silk cocoons can only provide degraded proteins, where the size and distribution of their molecular masses are significantly altered. Based on the plausible assumption that such effects can have a negative impact on the properties of sericin as a biomaterial, in this study we investigated comparatively four different extraction procedures in order to find the method that would cause the least hydrothermal degradation of BMSS. The products resulting from commonly used procedures (extraction in boiling water, alkaline extraction, and extraction in autoclave) were compared to those resulting from aqueous extraction in mild conditions as described a long time ago by Anderlini. The molecular mass distribution in BMSS resulting from each procedure was examined by electrophoretic analysis performed on sodium dodecyl sulphate-polyacrylamide gel (SDS-PAGE), while the conformational changes pertaining to secondary structure of BMSS were evaluated by Fourier transform infrared-attenuated total reflectance (FTIR-ATR) spectrometry. The electrophoretograms indicated that the aqueous extraction in mild conditions conducted at 50 °C for durations up to 4 weeks, with/without stirring, afforded the least degraded BMSS. The infrared spectrometric analysis showed that BMSS resulting from the mild extraction method contained predominantly β-sheet conformations, while the more degradative methods (alkaline, autoclave) led to BMSS where the random-coil conformations were preferential. The long-duration aqueous extraction at 50 °C (but not at 60 °C) appeared as a valid option for obtaining BMSS products where the hydrothermally induced fragmentation of the polypeptidic components is minimized.

## Introduction

Silks are natural proteinaceous composite materials constituted of assemblies of polypeptide and protein subunits, and they are generally classified as fibrous proteins. The polypeptidic assembly known as fibroin (henceforth BMSF), the main component of the silk threads produced by the larvae of domesticated silk moth (*Bombyx mori*), has become a promising biomaterial with relevant applications in tissue engineering and regenerative medicine (Altman et al. 2003; Vepari and Kaplan 2007; Hakimi et al. 2007; Wang et al. 2009; Murphy and Kaplan 2009; Harkin et al. 2011; Pritchard and Kaplan 2011; Wenk et al. 2011; Kundu et al. 2013; Hodgkinson and Bayat 2014; Kundu et al. 2014; Patra and Engel 2014; Koh et al. 2015; Kapoor and Kundu 2016). Sericin, the second major protein component of *B. mori* silk, has been acknowledged rather reluctantly as a potential biomaterial due to a hypothesized allergenic activity. In a previous study (Chirila et al. 2013), we not only have analyzed exhaustively the extant literature and concluded that this assertion was highly speculative and based on misinterpreted or misquoted results, but also have demonstrated experimentally that sericin can function as a substratum for the in vitro attachment and growth of a type of ocular cells (corneal limbal epithelial) without triggering any cytopathologic effect. Our results corroborated the evidence provided in previous or subsequent reports by other investigators (Minoura et al. 1995; Panilaitis et al. 2003; Tsubouchi et al. 2005; Terada et al. 2005; Xie et al. 2007; Teramoto et al. 2008; Aramwit et al. 2009a, b, 2010b; Hakimi et al. 2010; Aramwit 2014), which indicated that *B. mori* sericin (henceforth BMSS) should be regarded as a biocompatible material in spite of being traditionally associated with possible immune responses. Indeed, the general biocompatibility of BMSS was proved in many studies involving its direct contact with body cells or tissues, and consequently there has been an increased interest in using sericin-based products as biomaterials as shown in recent major publications (Kundu et al. 2008; Sehnal 2011; Khan and Tsukada 2014; Wang et al. 2014; Lamboni et al. 2015; Cao and Zhang 2016).

In our quest of developing silk-based biomedical materials, we were the first to evaluate the response of the eye’s cells or tissues to both BMSF (Chirila et al. 2008, 2016; Harkin et al. 2011; Madden et al. 2011; Shadforth et al. 2016) and BMSS (Chirila et al. 2013). Since BMSS proved to be a suitable substratum for the attachment of cells, we aim at extending the range of its applications as a biomaterial in tissue engineering, regenerative medicine, or surgery.

The assembly of polypeptides known as sericin represents about one-quarter of the total protein content of *B. mori* cocoons. The native BMSS acts as an adhesive coat for the pairs of triangular prismatic fibroin filaments (“brins”) and cements each pair into a single thread of silk. While fibroin is produced in the posterior silk gland of silkworms, sericin is produced in the middle silk gland and builds up three successive layers that envelop and glue the fibroin brins together (Kikkawa 1953; Tashiro and Otsuki 1970; Michaille et al. 1989; Inoue et al. 2000; Fedič et al. 2002; Julien et al. 2005). It has been established that native BMSF is composed of three protein subunits, H-chain, L-chain and glycoprotein P25 (fibrohexamerin), with the approximate molecular masses of, respectively, 360 kDa (but variously reported), 26 and 30 kDa, which are encoded by the *h*-*fib*, *l*-*fib* and *fhx* genes (Inoue et al. 2000; Julien et al. 2005). Unlike BMSF, the number of the subunits in BMSS is still disputed, and not much is known about their molecular mass either. Variable numbers of polypeptidic subunits in BMSS have been published over almost a century of research, such as two (Kondo 1921; Shelton and Johnson 1925; Kodama 1926), three (Mosher 1934; Rutherford and Harris 1940; Bryant 1948; Kikkawa 1953; Tashiro and Otsuki 1970; Takasu et al. 2002), five (Gamo et al. 1977), six (Tokutake 1980), or more (Sprague 1975; Fedič et al. 2002), while a range of molecular masses between 20 and 400 kDa has also been reported. Genomic analysis had so far indicated six genes (*Ser1*, *Ser2*, *Ser3*, *MSG*-*3*, *MSG*-*4*, and *MSG*-*5*) encoding for the BMSS secreted in the silkworm’s middle gland (Gamo 1982; Michaille et al. 1986, 1989; Grzelak 1995; Julien et al. 2005; Kundu et al. 2008), therefore we can expect at least six proteins to be present in the native BMSS. However, there is an important difference between the native BMSS and the processed (“regenerated”) BMSS, and it is not always clear whether the values previously reported for the distribution of molecular masses should be attributed to the native distribution of sericin proteins as secreted in the gland, or they reflect the distribution after isolation (extraction) of sericin from cocoons and further processing.

Procedures employed to isolate BMSF or BMSS from silk can cause the degradation (hydrolytic and/or thermal) of their constituent proteins, resulting in an increased number of fractions (oligopeptides, polypeptides), which consequently have markedly lower molecular masses as compared to those in their native state (i.e., in the silkworm’s gland or in the raw cocoon’s thread). An indirect proof of the effect of high temperatures on the degradation of BMSS proteins has been provided by its extraction from silk fibers through procedures performed at room temperature, using aqueous solutions of ethylenediamine/cupric hydroxide (Tokutake 1980) or lithium thiocyanate (Takasu et al. 2002) as solvents, when the electrophoresis revealed distinct polypeptide bands located at the high end of molecular mass range. It has been earlier suggested (Rutherford and Harris 1940) that native sericin is in fact a homogeneous protein, and the existence of more than one fraction in the product of the extraction procedure is an artifact caused by degradation. These investigators were wrong when proposing that BMSS in its native state was a single protein, but they were right in regard to potential effects of the hydrothermal degradative processes during isolation procedures. Such effects have been since confirmed by other investigators and will be discussed later in the present report.

## Rationale of the study

If regenerated BMSS is to become available on the shelf as a biomaterial, the preservation of the molecular mass size and distribution close to the native values appears desirable for a number of reasons. First, as a product for biomedical use, BMSS should be a unitary and reproducible material, and this can be assured by controlling the extent of chemical degradation during its isolation and processing. Fragmentation of the constituent proteins can alter the amino acid makeup leading to profound changes in the properties of resulting BMSS. Unavoidably, such changes would be strongly affected by the type and parameters of the extraction method. Second, the preservation of native molecular mass size and distribution may enhance the mechanical properties of BMSS-based substrata for tissue engineering (membranes, scaffolds, nanostructured constructs); for instance, we have shown (Chirila et al. 2013) that although the attachment of cells to BMSS membranes was superior to that to BMSF, the former membranes may be too flabby and weak for surgical manipulation. Third, the preservation of original molecular mass size and distribution may have a favorable effect on cytocompatibility and also may enhance the attachment and growth of cells. And lastly, it may contribute to the retention of the natural adhesive properties of native BMSS, which could elicit considerable interest in applications such as surgical adhesives.

On this background, the aim of the present investigation was to find a mild method for the isolation of BMSS, without involving potentially toxic solvents, with the expectation that its native molecular mass size and distribution will be less affected. In attempting this, we resurrected a long neglected method (Anderlini 1887a, b) and compared it to some of the current procedures.

## Materials

*B. mori* silkworm cocoons were supplied by Tajima Shoji Co Ltd. (Yokohama, Japan), with the pupae removed. Sericin *B. mori* powder was supplied by Sigma-Aldrich (St Louis, MO, USA) (cat. #S5201). All chemical reagents were also supplied by Sigma-Aldrich. Water of high purity (Milli-Q or equivalent) was used in all experiments. The Minisart^®^-GF pre-filters (0.7 μm) were supplied by Sartorius Stedim Biotech (Göttingen, Germany). The dialysis tubes with a molecular-mass cut-off (MMCO) at 3.5 kDa were supplied by Thermo Scientific (Rockford, IL, USA), and those with MMCO 12.4 kDa by Sigma-Aldrich. The concentrations and compositions in this report are expressed in percentage by weight, unless differently specified.

### Isolation of sericin

#### Extraction in boiling water

About 60 mg of *B. mori* silk cocoons were shredded into segments, and washed with water in a beaker by stirring for 30 min at room temperature. The washed materials were placed with 3 mL water in loosely screwed capped vials, and boiled for 10, 20 and 30 min, respectively. After filtration through a 0.7-μm Minisart^®^ filter, the resulting solutions were stored in closed containers at 60 °C (to prevent gelation) until subjected to electrophoretic analysis. Concentration of BMSS in the final solution was around 0.1 %, and the total extracted BMSS represented between 3 and 5 % from the initial silk cocoon weight.

#### Alkaline extraction

Cocoons (2.5 g) cut into 1 **×** 1 cm pieces were boiled in 1L of 0.02 M sodium carbonate solution for 1 h. After the insoluble fibers were removed, solution was filtered through a paper filter, and then concentrated to about 300 mL in a rotary evaporator (Rotavapor R-215, BÜCHI Labortechnik AG, Flawil, Switzerland). The concentrated solution was placed into dialysis tubing (MMCO 3.5 kDa) and dialyzed with stirring for 3 days while undergoing six water exchanges. The final solution was concentrated to about 30 mL, frozen at −80 °C and freeze-dried in a freeze dryer/vacuum concentrator (Alpha 1-2 LDplus, Martin Christ GmbH, Osterode, Germany). The powder, representing about 14 % of initial cocoon weight, was stored at room temperature until further use.

#### Extraction in autoclave

A published protocol (Nagura et al. 2001) was employed with some modifications. About 2 g of cocoon material, cut into 1 **×** 1 cm pieces, was washed at room temperature with water in a 2-L beaker with vigorous stirring for 30 min. The washed material was transferred to a screw-capped bottle loosely tightened, and containing 27 mL water, which was then placed in an autoclave and processed at 121 °C for various durations between 30 and 120 min, with or without subsequent dialysis. If is to be dialyzed, the resulting solution was filtered through a paper filter, and then injected into dialysis tubing (MMCO 12.4 kDa) that had been pre-treated by soaking in water for 4 h while exchanging the water five times. The tube was then placed in a 2-L beaker with hot water (80 °C) and dialyzed with stirring for 4 h, while maintaining the temperature and exchanging the water three times. The final solution was removed from the tube, filtered through a 0.7-μm Minisart^®^ filter, and collected in a closed glass container that was kept at 60 °C (to prevent gelation) until subsequent use. The concentration of BMSS in the solutions resulting from various experimental batches was determined by gravimetric analysis and found to be between 1 and 2 %. The total extracted BMSS represented about 9 % (autoclaving for 30 min followed by dialysis), and 14–17 % (autoclaving for 30–120 min, no dialysis) of the initial weight of cocoon material.

#### Aqueous extraction in mild conditions (Anderlini method)

While we followed the principle of this mild method as reported a long time ago (Anderlini 1887a, b), the subsequent processing was carried out using currently available techniques. About 1 g of shredded cocoons were placed in a screw-capped bottle, with 50 mL water containing 0.06 % sodium azide (for preventing bacterial growth), heated to either 50 or 60 °C in a temperature-controlled silicon oil bath, either with or without stirring, and maintained at the prescribed temperatures for 4 weeks. The changes with time were monitored by collecting 10-mL aliquots after 1, 2 or 3 weeks, respectively, followed by the addition of equivalent volumes of fresh water. At week 4, only the solid fibrous matter was removed (fibroin), while leaving the powdery precipitate deposited spontaneously on the bottom. This initial (“primary”) precipitate is assumed to contain sericin fractions that are insoluble due to higher molecular masses and/or structural effects. The supernatant solution, together with the primary precipitate, were placed in dialysis tubing (MMCO 3.5 kDa) and dialyzed under stirring for 3 days at 50 °C, with six water exchanges. The solution was then centrifuged at a RCF of 1000*g* for 20–30 min in an Eppendorf centrifuge (Model 5810 R) to separate the primary precipitate, which was subsequently freeze-dried to obtain a dry product. The supernatant phase was also freeze-dried leading to a powder (the “secondary” precipitate). This powder, together with the primary precipitate, was regraded as the total BMSS, and the sum of their weights represented about 8 % of the initial cocoon weight. The solid BMSS products were stored at room temperature prior to other use.

### Gel-electrophoretic analysis of extracted sericin

The molecular mass distribution of BMSS was investigated by sodium dodecyl sulphate-polyacrylamide gel electrophoresis (SDS-PAGE), using a Novex^®^ XCell Sure Lock™ Mini-Cell system (Life Technologies Inc, Carlsbad, CA, USA) and an EPS-250 Series II Power Supply unit (CBS Scientific Company Inc, San Diego, CA, USA).

Each BMSS solution resulting (a) either from the alkaline, autoclave or mild extraction procedures, and (b) either directly from extraction or from the dissolution in water of the solid products, was mixed with both Novex^®^ Tris–Glycine sample preparation reagent and NuPAGE^®^ sample reducing agent, and heated at 85 °C for 2 min. A volume of 10 μL BMSS solution containing about 50 μg protein matter was loaded into a 1-mm-thick 4–20 % Novex^®^ Tris–Glycine Gel in Novex^®^ Tris–Glycine SDS Running Buffer. The gels were run at a voltage of 125 V for 100 min together with the SeeBlue^®^ Pre-stained Protein Standard (Life Technologies, Australia). The resulting gel was washed in three 5-min stages with distilled water, and soaked in SimplyBlue™ SafeStain solution containing Coomassie^®^ G-250 stain for 1 h under gentle stirring. The gel was washed in distilled water for 1 h and then photographed using a Dolphin-Doc Plus system (Wealtec Corp, Sparks, NV, USA).

For comparison, a commercial sample of BMSS powder was dissolved in water and the solution (10 mg/mL) was also subjected to SDS-PAGE analysis in the conditions described above.

Based on our results in preliminary trials, in order to analyze the solutions that resulted from the extraction in boiling water, we have employed a slightly modified electrophoretic procedure, where the only differences consisted in that heating was done at 70 °C for 10 min, and that NuPAGE^®^ sample preparation reagent and 3–8 % NuPAGE^®^ Novex^®^ Tris–acetate gel in NuPAGE^®^ Tris–acetate SDS running buffer were used instead of the Tris–glycine equivalents. The resulting gels were run at a voltage of 150 V for 1 h together with a HiMark™ Pre-stained Protein Standard (Life Technologies, Australia).

### Analysis by Fourier transform infrared-attenuated total reflectance (FTIR-ATR) spectrometry

FTIR-ATR spectra of selected BMSS samples were collected and recorded using a Nicolet Nexus 5700 FTIR spectrometer (Thermo Electron Corp, Marietta, OH, USA), equipped with a Nicolet Smart Endurance diamond ATR accessory. Each spectrum was obtained by co-adding 64 scans in the range from 4000 to 525 cm^−1^, at a resolution of 8 cm^−1^. For recording and analyzing the spectra, the OMNIC 7 software package (Thermo Electron Corp) was used.

## Results

### Molecular mass distribution

**Fig. 1:**
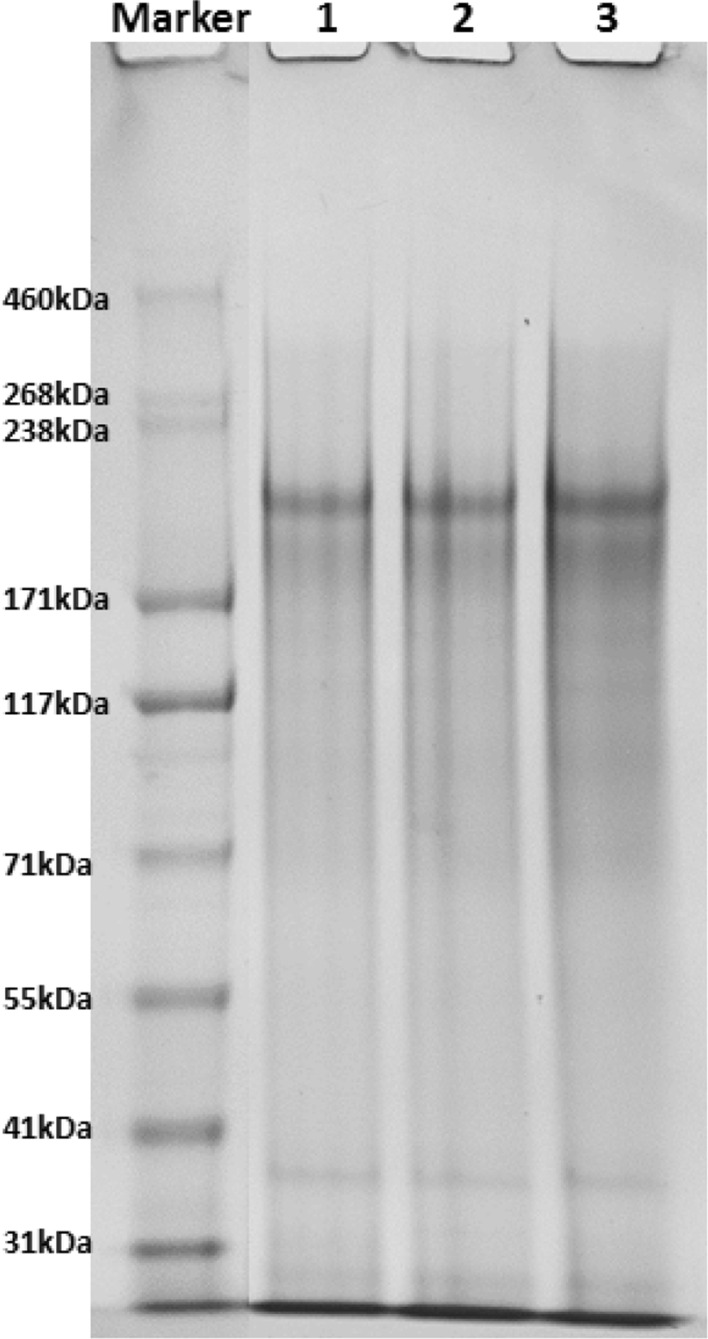
Electrophoretic patterns of BMSS extracted in boiling water (*lanes* *1*–*3*). *Left lane* shows the molecular mass marker positions. The *numbers* represent duration of boiling: *1*—10 min; *2*—20 min; *3*—30 min. Identical amounts of the samples were loaded for electrophoresis. See text for details of the analysis

The electrophoretic mobility of the BMSS components obtained by the four extraction procedures was investigated by SDS-PAGE with the aim of visualizing the distribution of their molecular masses. Figure 1 shows the electrophoretic pattern displayed by the BMSS solution resulted from extraction in boiling water for relatively short durations. The highest molecular mass attributable to a distinct protein was around 200 kDa, while less defined bands were observable up to the region around 300 kDa. As expected, the smear patterns became more obvious as the duration of boiling increased.

**Fig. 2:**
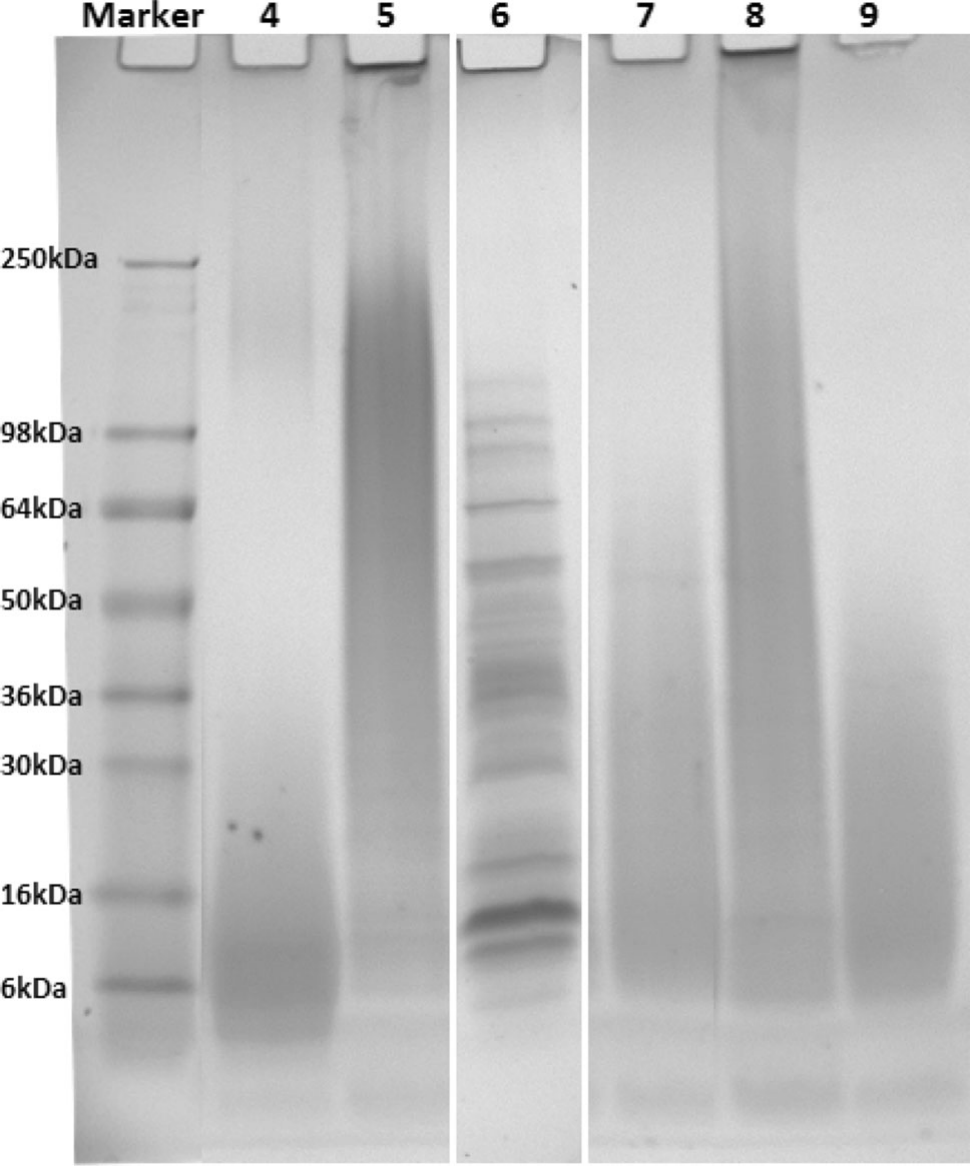
Electrophoretic patterns of BMSS extracted by various procedures (*lanes* *4*–*9*). *Left lane* shows the molecular mass marker positions. The *numbers* represent procedures and samples: *4* alkaline extraction; *5* extraction in autoclave (30 min); *6* aqueous extraction in mild conditions (50 °C/4 weeks/no stirring): fractions in the supernatant (secondary precipitate); *7* aqueous extraction in mild conditions (60 °C/4 weeks/with stirring): fractions in the supernatant; *8* aqueous extraction in mild conditions (60 °C/4 weeks/with stirring): fractions in the primary precipitate; *9* dissolved commercial sericin sample. Identical amounts of the samples were loaded for electrophoresis. See text for details of the analysis

Figure 2 (lane *4*) revealed that the alkaline extraction had the most pronounced degradative effect on BMSS, as illustrated by an undefined smear front below ~35 kDa, although a minor distribution was vaguely apparent around 200 kDa. The electrophoretic distribution of BMSS isolated by extraction in autoclave is shown in Fig. 2 (lane *5*). A contiguous smear pattern covering a large array of molecular masses (between 10 and 250 kDa) indicated significant hydrothermal degradation resulting in a large number of polypeptides, while proteins with higher molecular mass are still present.

The electrophoretic pattern of BMSS extracted in mild conditions (50 °C in water, for 4 weeks, without stirring) is shown in Fig. 2 (lane *6*), regarding the material that was precipitated through freeze-drying from the initial supernatant liquid (i.e., the secondary precipitate). A relatively large number of distinct polypeptidic fragments between 10 and 100 kDa indicated a moderate degradation. As this fraction consisted of soluble polypeptides, the absence of components with higher molecular mass is self-explainable. Upon rising the temperature by only 10° and using stirring, the same extraction procedure led to significant degradation, both in the product that was precipitated by freeze-drying from the initial supernatant (Fig. 2, lane *7*) and in the initial spontaneous precipitate, what we called the primary precipitate (Fig. 2, lane *8*). However, polypeptidic fragments with higher molecular masses (in excess of ~300 kDa), therefore insoluble in water, were abundant in the latter but poorly defined. As a comparison and a test of our electrophoretic analysis, the solution of a commercially available sericin sample, supplied by Sigma-Aldrich, was also analyzed, resulting in the pattern shown in Fig. 2 (lane *9*). Clearly, this product was significantly degraded during isolation procedure, and the molecular mass distribution (10–40 kDa) suggests that alkaline extraction was the method used for its isolation.

**Fig. 3:**
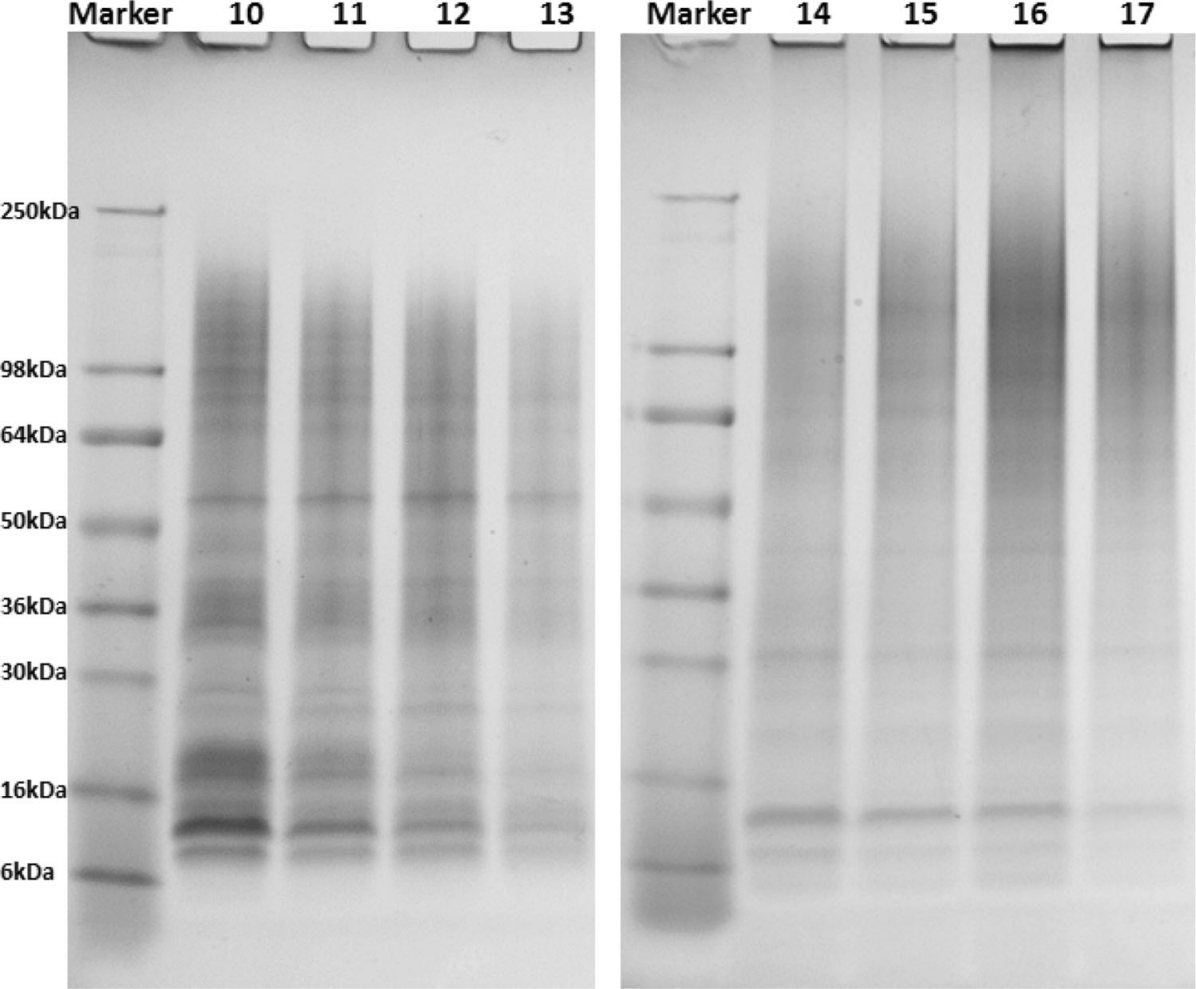
Influence of duration on the electrophoretic patterns of BMSS extracted in mild conditions at 50 °C for up to 4 weeks, with stirring: fractions in the initial supernatant isolated by freeze-drying as the secondary precipitate (*lanes* *10*–*13*) and fractions in the primary precipitate (*lanes* *14*–*17*). *Left lane* shows the molecular mass marker positions. The *numbers* represent the duration of extraction: *10* and *14*—1 week; *11* and *15*—2 weeks; *12* and *16*—3 weeks; *13* and *17*—4 weeks. Identical amounts of the samples were loaded for electrophoresis. See text for details of the analysis

**Fig. 4:**
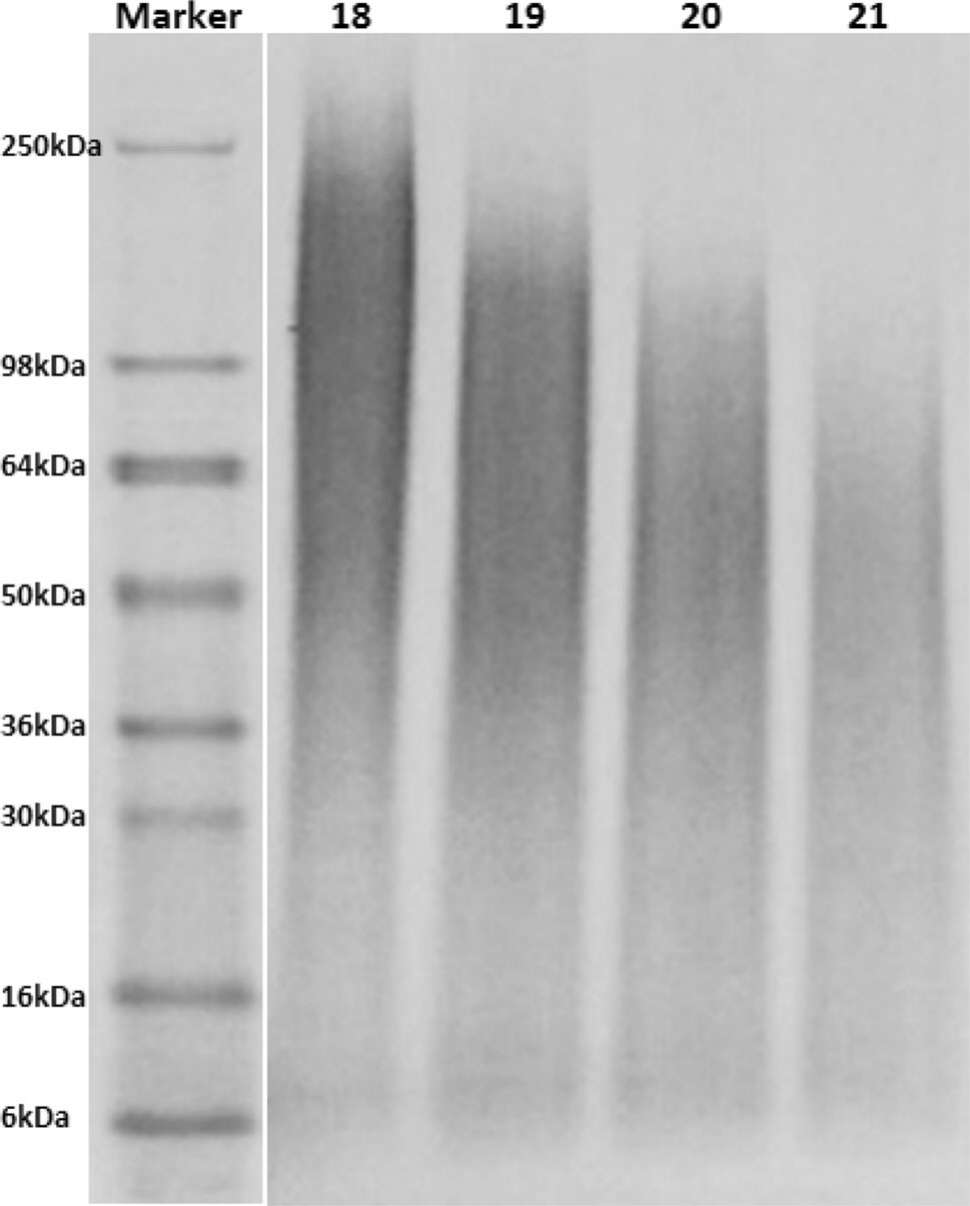
Influence of duration on the electrophoretic patterns of BMSS extracted in autoclave at 121 °C without subsequent dialysis. *Left lane* shows the molecular mass marker positions. The *numbers* represent the duration of extraction: *18*—30 min; *19*—60 min; *20*—90 min; *21*—120 min. Identical amounts of the samples were loaded for electrophoresis. See text for details of the analysis. Compare *lane* *18* with *lane* *5* (Fig. 2), corresponding to dialyzed material

The effect of duration of the mild extraction process on the electrophoretic patterns is shown in Fig. 3. While the BMSS isolated from the initial supernatant (lanes *10*–*13*) showed a decrease in the amount of polypeptides with high molecular mass as the duration of the process increased, the distribution of molecular mass in the primary precipitate (lanes *14*–*17*) was little affected by longer durations. The effect of duration was even more dramatic in the case of the autoclave extraction as seen in Fig. 4 (lanes *18*–*21*). It can be noticed that the electrophoretogram in lane *5* (Fig. 2), corresponding to extraction in autoclave (30 min) followed by dialysis, is practically identical to that in lane *18* (Fig. 4), corresponding to extraction in the same conditions, but without being subjected to dialysis. This finding may cast doubt on the necessity of dialysis, which indeed can remove the contaminants with low molecular mass, but apparently does not affect the number and distribution of the sericinoid polypeptides in the extracted BMSS.

### Effects on the secondary structure of BMSS

The FTIR-ATR spectra of the samples extracted in different conditions showed significant differences in the secondary structure of the BMSS products resulted from extraction in mild conditions (Anderlini method) and those resulting from extraction in degradative conditions (alkaline, autoclave).

## Discussion

An operation known as “degumming” is used on industrial scale to remove the sericin coating (“gum”) from *B. mori* silk fibers. It is a mandatory stage in the processing of silk textiles, and is generally performed before the dying operation, to ultimately render the sericin into an industrial waste. Current methods for degumming include treatment with soaps or other alkaline agents, high temperature/high pressure treatment, boiling in water, enzymatic degradation, and treatment with organic acids. The recovery of BMSS from the residual “baths” has been attempted, but appeared to be economically unfeasible. Also, the resulting sericin is degraded to the extent that little resemblance to the native BMSS remained. For instance, following degumming experiments with several proteolytic enzymes, it was found that the range of molecular weight of the resulting mixture of sericinoid polypeptides dropped to values between 5 and 20 kDa (Freddi et al. 2003). One can legitimately conclude that if we need to obtain BMSS with values for the size and distribution of molecular mass similar to the native values, we shall isolate BMSS directly from the silkworm’s middle gland. Unfortunately, this procedure would be utterly impractical for obvious reasons. Besides, it is unlikely that the resulting material will be uniform, and also would need to be further purified because of contamination with other natural compounds, such as pigments (carotenoids, flavonoids), fats, paraffins, uric acid, and proteinase inhibitors (Bergmann 1938a, b, 1939; Kurioka et al. 1999; Kurioka and Yamazaki 2002a, b; Nirmala et al. 2001). While some of these “contaminants” can be useful in biomedical applications due to inherent antioxidative or antimicrobial properties, they do not outlast the isolation procedures, either due to chemical modification caused by hydrothermal conditions at extraction or to physical removal during subsequent purification.

Although not aimed at isolating the sericin, but rather at its complete removal as debris, the degumming technology played nevertheless a significant role in the conceptual development of sericin as a major component of silk. It prompted the earliest report on sericin in 1785, when l’Abbé (“Father”) Collomb in France was the first to show that sericin, then called *“vernis de la soie”* (“varnish of the silk”) or *“naturel enduit de la soie”* (“natural coating of the silk”), can be easily removed in boiling water (Collomb 1785), such contradicting the prevailing dogma that silk coating can be dissolved only in soap or alkaline solutions. Most notably, Collomb realized that water vapors under pressure will be even more effective than the boiling water at atmospheric pressure. To demonstrate this, he used the “Papin digester”, the precursor of the autoclave that was invented by Denis Papin during the previous century (Papin 1681). It is enthralling that one of the earliest uses of the autoclave in scientific research was to extract sericin, perhaps reflecting the interest-driven support from silk textile manufacturers. Collomb’s discovery was highly appreciated, and an academic committee in city of Lyon attended his degumming experiments carried in a custom-built digester of very large capacity. A report published at that time (Encylopédie 1790) hailed Collomb’s work as an achievement of great importance and utility, and estimated that about 120 tonnes of soap could be annually saved by his method. Not much later, the attention of some scientists became focused toward research on sericin as such, and the need for studying in more depth the extraction process became evident. An early study (Roard 1808) on the effects of water, alcohol, alkaline solutions, soap, and acids on the silk yarn identified two fractions that were assumed to be constituents of the gum coating. Improved methods to remove and isolate sericin with the sole purpose of investigating its composition and structure have been explored throughout 19th and 20th centuries, as shown in some earlier reviews (Shelton and Johnson 1925; Lucas et al. 1958). During this classic era of sericin research, the extraction in boiling water (Cramer 1865; Wetzel 1899; Bondi 1902) and by autoclaving (Fischer and Skita 1902; Kondo 1921; Shelton and Johnson 1925; Kodama 1926) became established techniques for isolation of sericin from cocoons or raw silk.

Anderlini’s research was triggered by his general observation that the materials of animal origin are affected profoundly when treated in boiling water, and in particular that sericin extracted in boiling water for a short time is different form the one isolated after lengthy boiling. Therefore, he decided to investigate what happens at low temperatures (Anderlini 1887a). The experiments at room temperature had to be abandoned because of massive bacterial growth. Most of extractions were then carried out between 50 and 60 °C, for durations between 12 h and 2 months, with intermittent squeezing of the cocoon material in a winepress. The collected solutions were concentrated subsequently at 80 °C or by boiling, which indicates that Anderlini was not actually sure about the effect of hydrothermal degradation, although he was aware that high temperatures might induce some structural changes in sericin. At the time of Anderlini’s experiments, the concept of molecular mass was relatively new. The proteins were identified as class of natural compounds toward the end of the eighteenth century, and included substances such as albumin or blood plasma’s fibrin. However, little progress in this field has been seen during the nineteenth century because of difficulty in purifying and analyzing such compounds; in fact, the polypeptidic nature of proteins was revealed about two decades after Anderlini’s reports. Employing a rather complex succession of precipitations with inorganic salts, filtrations, removal of the inorganic precipitates, re-precipitations of the organic phases with ethanol, re-filtrations, etc., Anderlini managed to isolate three distinct fractions from sericin that he called α, β and γ, respectively. In a subsequent report, he concluded that only fraction γ should be regarded as true sericin (Anderlini 1887b). However, when he subjected the three fractions to a burn test, all samples produced the same odor specific to proteinaceous materials. This rather invalidates his conclusion and suggests that these fractions were all sericinoid polypeptides with different ranges of molecular masses.

Comparative investigations on various methods for the extraction of sericin have been also reported in more recent times. Employing sericin produced by a *B. mori* mutant race known as “Sericin Hope”, a silkworm that does not synthesize fibroin, it was found that the fractions collected directly from the cocoons’ fiber (Teramoto et al. 2005) or from the glands (Teramoto et al. 2006) displayed an electrophoretic pattern comprising at least five distinct polypeptide bands. The same sericin, when extracted in autoclave, showed an undefined smear pattern. Sericin isolated from a fibroin-deficient *B. mori* mutant strain (*185 Nd*-*s*) by extraction at 35 °C in lithium bromide solution displayed four distinct bands between 60 and ~300 kDa, while the product extracted by alkaline boiling showed a smear pattern from ~10 to ~250 kDa (Wang et al. 2014). The autoclave method as such has been thoroughly investigated in normal *B. mori* silk between 82 and 120 °C for various durations (Sothornvit et al. 2010); a statistic treatment of the experimental process variables indicated 115 °C for 37 min as the optimum set of parameters for extracting BMSS, but even in these conditions the poorly defined electrophoretic bands were scattered over a region between ~50 and ~130 kDa, suggesting significant degradation. An in-depth comparative study of four different extraction procedures showed that molecular mass size and distribution were not the only characteristics affected by the extraction conditions (Aramwit et al. 2010a, b). Comparing the autoclave, alkaline, citric acid, and urea methods, the investigators found that the first three methods led to degraded products that display diffuse electrophoretic patterns with very few, poorly defined bands. The urea extraction method was the only method that afforded products with a large number of distinct electrophoretic bands up to ~300 kDa. Unfortunately, the resulting sericin contained residual urea that could not be removed by dialysis (Aramwit et al. 2010a), and the urea-contaminated sericin displayed significant cytotoxicity in cell culture experiments (Aramwit et al. 2010b). Alternative extraction methods affected differently certain properties of the resulting sericins such as secondary structure, amino acid composition, thermal behavior, or antityrosinase activity (Aramwit et al. 2010a), as well as surface charge, and viability of cells cultivated on sericins (Aramwit et al. 2010b). Other investigators (Kurioka et al. 2004) have also found that some biochemical properties of sericin (in this case the trypsin inhibitory effect) could be affected by the type of extraction process.

Our results in this study confirm in part the findings of these investigators. Clearly, the higher the temperature and the longer the duration of extraction process, the more advanced the degradation of the sericinoid proteins. As expected, the alkaline extraction caused the most extensive degradation (Fig. 2, lane *4*). If the duration of the process is short, a high temperature may elicit less degradation, as seen in the case of short-term boiling in neutral water (Fig. 1), but a quantitative isolation of all components seems unlikely. Our results also showed that Anderlini’s mild procedure caused minimum degradation when performed at 50 °C for 1–4 weeks as seen in Fig. 2 (lane *6*) and Fig. 3. Comparing the electrophoretogram in lane *6* (Fig. 2) with that in lane *13* (Fig. 3), it is obvious that stirring enhances the degradative process, and actually the duration of extraction at 50 °C should be no longer than 3 weeks when is accompanied by stirring. When using this method, we also have to differentiate between the polypeptidic fractions precipitated during the process (the primary precipitate) and those precipitated subsequently from the supernatant liquid phase by freeze-drying (the secondary precipitate), the latter corresponding to lanes *10*–*13* in Fig. 3. Lanes *14*–*17* in the same figure indicated that the primary precipitate contained mainly high molecular mass components, which were degraded to a larger extent than those in the secondary precipitate. An interesting finding was the rather dramatic enhancement of the hydrothermal degradation when the extraction was performed at 60 °C as shown in Fig. 2 (lanes *7* and *8*), which suggests the existence of a threshold temperature. We can only be appreciative of Anderlini’s foresight when he advised that the temperature should not overstep 60 °C (*“… senza però oltrepassare i 60°”*) (Anderlini 1887a, p 313), a remarkable intuition considering that no techniques were available at that time to enable monitoring the effect of temperature on hydrothermal degradation and the ensuing molecular mass re-distribution.

**Fig. 5:**
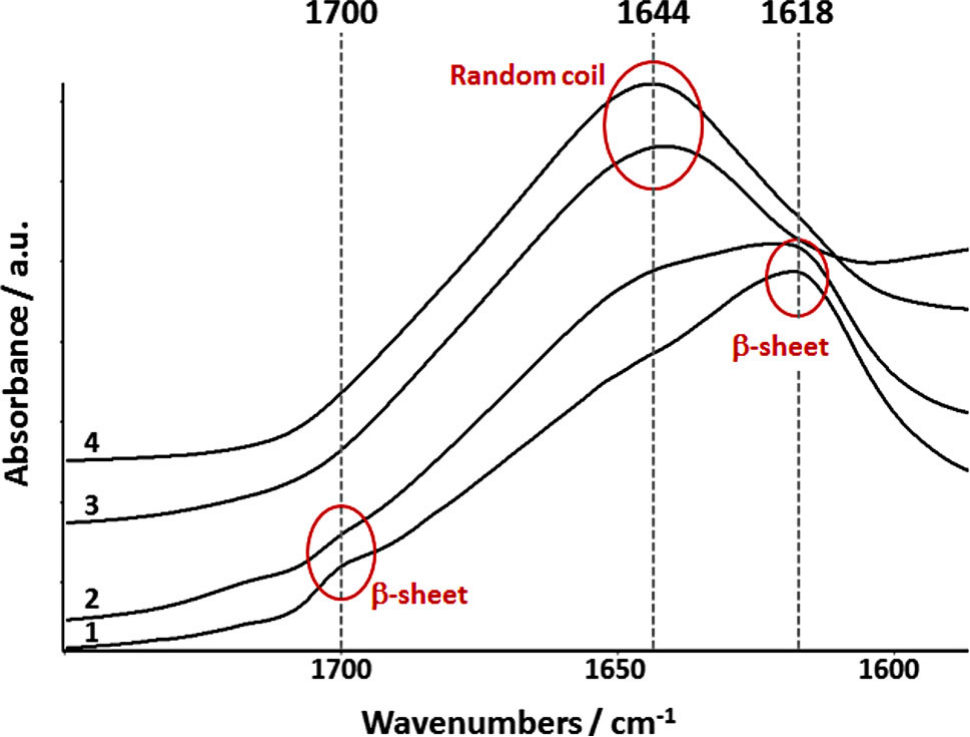
The “Amide I” region in the FTIR-ATR spectra of BMSS extracted by various methods. The numbers represent the method of extraction and the resulting BMSS samples: *1*—BMSS in the primary precipitate, Anderlini method (50 °C/4 weeks/with stirring), corresponding to *lane* *17* in Fig. 3; *2*—BMSS in the secondary precipitate, Anderlini method (50 °C/4 weeks/with stirring), corresponding to *lane* *13* in Fig. 3; *3*—BMSS extracted by alkaline method, corresponding to *lane* *4* in Fig. 2; *4*—BMSS extracted in autoclave (121 °C/30 min), corresponding to *lane* *5* in Fig. 2

The infrared spectrum in the “Amide I” region (from ~1590 cm^−1^ to ~1720 cm^−1^) is routinely used for the characterization of the secondary structure of BMSF or BMSS. In Fig. 5 (spectra *1* and *2*), it can be seen that both BMSS precipitates resulting by Anderlini extraction method (50 °C/4 weeks/with stirring) displayed an intensive band at 1618 cm^−1^ as well as a shoulder at 1700 cm^−1^, both absorptions indicating the predominance of β-sheet conformations and their aggregates (Teramoto and Miyazawa 2005; Teramoto et al. 2005, 2008; Chirila et al. 2013), suggesting high crystallinity. On the contrary, the spectra of the BMSS samples extracted by either alkaline method (Fig. 5, spectrum *3*) or in autoclave (Fig. 5, spectrum *4*) exhibited a strong absorption band at 1644 cm^−1^, indicating the predominance of random-coil conformation (Lee et al. 2003; Aramwit et al. 2010a). Our findings confirm that the method of extraction of BMSS can induce significant changes in the secondary of BMSS, which perhaps are related to hydrolytic degradative reactions and/or to effects of the processing temperature.

## Conclusion

In order to prevent the advanced hydrothermal degradation of the constituent proteins in *B. mori* silk sericin during the extraction process, and aiming at obtaining products with potentially better biomaterial properties, a mild aqueous extraction of longer duration carried out at 50 °C is an effective alternative to the current extraction procedures.

### **Authors’ contributions**

TVC: designed and coordinated the study; contributed to the interpretation of results; wrote the manuscript. SS: carried out the production and analysis of silk sericin materials; contributed to the study design and to the interpretation of results; drafted sections of the manuscript; executed and organized the graphic material in the manuscript; reviewed the final draft of the manuscript. NCM: executed parts of the experiments; contributed to the interpretation of results; reviewed the final draft of the manuscript.

## Abbreviations

BMSF: *Bombyx mori* silk fibroin
BMSS: *Bombyx mori* silk sericin
FTIR-ATR: Fourier transform infrared-attenuated total reflectance
MMCO: Molecular-mass cut-off
SDS-PAGE: Sodium dodecyl sulphate-polyacrylamide gel electrophoresis

## References

[CR1] Altman GH, Diaz F, Jakuba C, Calabro T, Horan RL, Chen J, Lu H, Richmond J, Kaplan DL (2003). Silk-based biomaterials. Biomaterials.

[CR2] Anderlini F (1887). Ricerche chimiche sulla seta (chemical investigations on the silk). Atti Reale Ist Veneto Sci Lett Arti (Serie 6).

[CR3] Anderlini F (1887). Ricerche chimiche sulla seta (chemical investigations on the silk). Atti Reale Ist Veneto Sci Lett Arti (Serie 6).

[CR4] Aramwit P, Kundu SC (2014). Bio-response to silk sericin. Silk biomaterials for tissue engineering and regenerative medicine.

[CR5] Aramwit P, Kanokpanont S, De-Eknamkul W, Kamei K, Srichana T (2009). The effect of sericin with variable amino-acid content from different silk strains on the production of collagen and nitric oxide. J Biomater Sci Polym Ed.

[CR6] Aramwit P, Kanokpanont S, De-Eknamkul W, Srichana T (2009). Monitoring of inflammatory mediators induced by silk sericin. J Biosci Bioeng.

[CR7] Aramwit P, Damrongsakkul S, Kanokpanont S, Srichana T (2010). Properties and antityrosinase activity of sericin from various extraction methods. Biotechnol Appl Biochem.

[CR8] Aramwit P, Kanokpanont S, Nakpheng T, Srichana T (2010). The effect of sericin from various extraction methods on cell viability and collagen production. Int J Mol Sci.

[CR9] Bergmann W (1938). The distribution of wax in cocoon silk. Textile Res.

[CR10] Bergmann W (1938). A study of the composition of the ether soluble fraction of raw silk. Textile Res.

[CR11] Bergmann W (1939). The natural pigments of silk. Textile Res.

[CR12] Bondi S (1902). Studien über der Seidenleim (studies on sericin). Hoppe-Seyler’s Zeitschr Physiol Chem.

[CR13] Bryant F (1948). The sericin fractions of raw silk. Textile J Aust.

[CR14] Cao T-T, Zhang Y-Q (2016). Processing and characterization of silk sericin from *Bombyx mori* and its application in biomaterials and biomedicines. Mater Sci Eng C.

[CR15] Chirila TV, Barnard Z, Zainuddin Harkin DG, Schwab IR, Hirst LW (2008). *Bombyx mori* silk fibroin membranes as potential substrata for epithelial constructs used in the management of ocular surface disorders. Tissue Eng A.

[CR16] Chirila TV, Suzuki S, Bray LJ, Barnett NL, Harkin DG (2013). Evaluation of silk sericin as a biomaterial: in vitro growth of human corneal limbal epithelial cells on *Bombyx mori* sericin membranes. Prog Biomater.

[CR17] Chirila TV, Suzuki S, Hirst LW, Harkin DG, Chirila TV, Harkin DG (2016). Reconstruction of the ocular surface using biomaterial templates. Biomaterials and regenerative medicine in ophthalmology.

[CR18] l’Abbé Collomb (1785) Observations sur la dissolution du vernis de la soie (observations on the dissolution of the varnish of the silk). Obs Phys Hist Nat Arts 27(II):95–107

[CR19] Cramer E (1865). Ueber die Bestandtheile der Seide (on the constituents of silk). J Prakt Chem.

[CR20] Encyclopédie méthodique (1790) Manufactures, arts et métiers. Errata, supplement, et vocabulaire, de la premiere partie complétant le Tome Second. Panckoucke, Paris, pp 27–34

[CR21] Fedič R, Žurovec M, Sehnal F (2002). The silk of Lepidoptera. J Insect Biotechnol Sericol.

[CR22] Fischer E, Skita A (1902). Ueber das Fibroin und den Leim der Seide (on fibroin and sericin). Hoppe-Seyler’s Zeitschr Physiol Chem.

[CR23] Freddi G, Mossotti R, Innocenti R (2003). Degumming of silk fabric with several proteases. J Biotechnol.

[CR24] Gamo T (1982). Genetic variants of the *Bombyx mori* silkworm encoding sericin proteins of different lengths. Biochem Genet.

[CR25] Gamo T, Inokuchi T, Laufer H (1977). Polypeptides of fibroin and sericin secreted from the different sections of the silk gland. Insect Biochem.

[CR26] Grzelak K (1995). Control of expression of silk protein genes. Comp Biochem Physiol.

[CR27] Hakimi O, Knight DP, Vollrath F, Vadgama P (2007). Spider and mulberry silkworm silks as compatible biomaterials. Compos B.

[CR28] Hakimi O, Gheysens T, Vollrath F, Grahn MF, Knight DP, Vadgama P (2010). Modulation of cell growth on exposure to silkworm and spider silk fibers. J Biomed Mater Res.

[CR29] Harkin DG, George KA, Madden PW, Schwab IR, Hutmacher DW, Chirila TV (2011). Silk fibroin in ocular tissue reconstruction. Biomaterials.

[CR30] Hodgkinson AT, Bayat A, Kundu SC (2014). Silk for dermal tissue engineering. Silk biomaterials for tissue engineering and regenerative medicine.

[CR31] Inoue S, Tanaka K, Arisaka F, Kimura S, Ohtomo K, Mizuno S (2000). Silk fibroin of *Bombyx mori* is secreted, assembling a high molecular mass elementary unit consisting of H-chain, L-chain, and P25, with a 6:6:1 molar ratio. J Biol Chem.

[CR32] Julien E, Coulon-Bublex M, Garel A, Royer C, Chavancy G, Prudhomme J-C, Couble P, Gilbert LI, Iatrou K, Gill SS (2005). Silk gland development and regulation of silk protein genes. Comprehensive molecular insect science.

[CR33] Kapoor S, Kundu SC (2016). Silk protein-based hydrogels: promising advanced materials for biomedical applications. Acta Biomater.

[CR34] Khan MMR, Tsukada M, Kundu SC (2014). Electrospun silk sericin nanofibers for biomedical applications. Silk biomaterials for tissue engineering and regenerative medicine.

[CR35] Kikkawa H (1953). Biochemical genetics of *Bombyx mori* (silkworm). Adv Genetics.

[CR36] Kodama K (1926). The preparation and physico-chemical properties of sericin. Biochem J.

[CR37] Koh L-D, Cheng Y, Teng C-P, Khin Y-W, Loh X-J, Tee S-Y, Low M, Ye E, Yu H-D, Zhang Y-W, Han M-Y (2015). Structures, mechanical properties and applications of silk fibroin materials. Prog Polym Sci.

[CR38] Kondo K (1921). Research on some properties of sericin. J Chem Soc Japan (Nippon Kagaku Kaishi).

[CR39] Kundu SC, Dash BC, Dash R, Kaplan DL (2008). Natural protective glue protein, sericin bioengineered by silkworms: potential for biomedical and biotechnological applications. Prog Polym Sci.

[CR40] Kundu B, Rajkhowa R, Kundu SC, Wang X (2013). Silk fibroin biomaterials for tissue regenerations. Adv Drug Deliv Rev.

[CR41] Kundu B, Kurland NE, Bano S, Patra C, Engel FB, Yadavalli VK, Kundu SC (2014). Silk proteins for biomedical applications: bioengineering perspectives. Prog Polym Sci.

[CR42] Kurioka A, Yamazaki M (2002). Purification and identification of flavonoids from the yellow green cocoon shell (Sasamayu) of the silkworm, *Bombyx mori*. Biosci Biotechnol Biochem.

[CR43] Kurioka A, Yamazaki M (2002). Antioxidant in the cocoon of the silkworm, *Bombyx mori*. J Insect Biotechnol Sericol.

[CR44] Kurioka A, Yamazaki M, Hirano H (1999). Primary structure and possible functions of a trypsin inhibitor in *Bombyx mori*. Eur J Biochem.

[CR45] Kurioka A, Kurioka F, Yamazaki M (2004). Characterization of sericin powder prepared from citric acid-degraded sericin polypeptides of the silkworm, *Bombyx mori*. Biosci Biotechnol Biochem.

[CR46] Lamboni L, Gauthier M, Yang G, Wang Q (2015). Silk sericin: a versatile material for tissue engineering and drug delivery. Biotechnol Adv.

[CR47] Lee K, Kweon H, Yeo JH, Woo SO, Lee YW, Cho C-S, Kim KH, Park YH (2003). Effect of methyl alcohol on the morphology and conformational characteristics of silk sericin. Int J Biol Macromol.

[CR48] Lucas F, Shaw JTB, Smith SG (1958). The silk fibroins. Adv Protein Chem.

[CR49] Madden PW, Lai JNX, George KA, Giovenco T, Harkin DG, Chirila TV (2011). Human corneal endothelial cell growth on a silk fibroin membrane. Biomaterials.

[CR50] Michaille JJ, Couble P, Prudhomme J-C, Garel A (1986). A single gene produces multiple sericin messenger RNAs in the silk gland of *Bombyx mori*. Biochimie.

[CR51] Michaille JJ, Garel A, Prudhomme JC (1989). The expression of five middle silk gland specific genes is territorially regulated during the larval development of *Bombyx mori*. Insect Biochem.

[CR52] Minoura N, Aiba S, Gotoh Y, Tsukada M, Imai Y (1995). Attachment and growth of cultured fibroblast cells on silk protein matrices. J Biomed Mater Res.

[CR53] Mosher H (1934). The sericin fractions of silk. Am Silk Rayon J.

[CR54] Murphy AR, Kaplan DL (2009). Biomedical applications of chemically-modified silk fibroin. J Mater Chem.

[CR55] Nagura M, Ohnishi R, Gitoh Y, Ohkoshi Y (2001). Structures and physical properties of cross-linked sericin membranes. J Insect Biotechnol Sericol.

[CR56] Nirmala X, Kodrik D, Žurovec M, Sehnal F (2001). Insect silk contains both a Kunitz-type and a unique Kazal-type proteinase inhibitor. Eur J Biochem.

[CR57] Panilaitis B, Altman GH, Chen J, Jin H-J, Karageorgiou V, Kaplan DL (2003). Macrophage responses to silk. Biomaterials.

[CR58] Papin D (1681). A new digester or engine for softning bones, containing the description of its make and use in these particulars: viz. cookery, voyages at sea, confectionary, making of drinks, chymistry, and dying.

[CR59] Patra C, Engel FB, Kundu SC (2014). Silk for cardiac tissue engineering. Silk biomaterials for tissue engineering and regenerative medicine.

[CR60] Pritchard EM, Kaplan DL (2011). Silk fibroin biomaterials for controlled release drug delivery. Expert Opin Drug Deliv.

[CR61] Roard J-L (1808). Mémoire sur le décreusage de la soie (dissertation on the degumming of the silk). Ann Chim.

[CR62] Rutherford HA, Harris M (1940). Concerning the existence of fractions of the sericin in raw silk. Textile Res.

[CR63] Sehnal F (2011) Biotechnologies based on silk. In: Vilcinskas A (ed) Insect biotechnology. Gorb SN (ed) Biologically-inspired systems series, vol 2. Springer, Dordrecht, pp 211–224

[CR64] Shadforth AMA, Chirila TV, Harkin DG, Kwan ASL, Chen FK, Chirila TV, Harkin DG (2016). Biomaterial templates for the culture and transplantation of retinal pigment epithelial cells: a critical review. Biomaterials and regenerative medicine in ophthalmology.

[CR65] Shelton EM, Johnson TB (1925). Research on proteins. VII. The preparation of the protein “sericin” from silk. J Am Chem Soc.

[CR66] Sothornvit R, Chollakup R, Suwanruji P (2010). Extracted sericin from silk waste for film formation. Songklanakarin J Sci Technol.

[CR67] Sprague KU (1975). The *Bombyx mori* silk proteins characterization of large polypeptides. Biochemistry.

[CR68] Takasu Y, Yamada H, Tsubouchi K (2002). Isolation of three main sericin components from the cocoon of the silkworm, *Bombyx mori*. Biosci Biotechnol Biochem.

[CR69] Tashiro Y, Otsuki E (1970). Studies on the posterior silk gland of the silkworm *Bombyx mori*. J Cell Biol.

[CR70] Terada S, Sasaki M, Yanagihara K, Yamada H (2005). Preparation of silk protein sericin as mitogenic factor for better mammalian cell culture. J Biosci Bioeng.

[CR71] Teramoto H, Miyazawa M (2005). Molecular orientation behaviour of silk sericin as revealed by ATR infrared spectroscopy. Biomacromolecules.

[CR72] Teramoto H, Nakajima K, Takabayashi C (2005). Preparation of elastic sericin hydrogel. Biosci Biotechnol Biochem.

[CR73] Teramoto H, Kakazu A, Asakura T (2006). Native structure and degradation pattern of silk sericin studied by ^13^C NMR spectroscopy. Macromolecules.

[CR74] Teramoto H, Kameda T, Tamada Y (2008). Preparation of gel film from *Bombyx mori* silk sericin and its characterization as a wound dressing. Biosci Biotechnol Biochem.

[CR75] Tokutake S (1980). Isolation of smallest component of silk protein. Biochem J.

[CR76] Tsubouchi K, Igarashi Y, Takasu Y, Yamada H (2005). Sericin enhances attachment of cultured human skin fibroblasts. Biosci Biotechnol Biochem.

[CR77] Vepari C, Kaplan DL (2007). Silk as a biomaterial. Prog Polym Sci.

[CR78] Wang X, Cebe P, Kaplan DL, Lutz S, Bornscheuer UT (2009). Silk proteins—biomaterials and engineering. Protein engineering handbook.

[CR79] Wang Z, Zhang Y, Zhang J, Huang L, Liu J, Li Y, Zhang G, Kundu SC, Wang L (2014). Exploring natural silk protein sericin for regenerative medicine: an injectable, photoluminescent, cell-adhesive 3D hydrogel. Sci Rep.

[CR80] Wenk E, Merkle HP, Meinel L (2011). Silk fibroin as a vehicle for drug delivery applications. J Control Rel.

[CR81] Wetzel G (1899). Ein Beitrag zur Kenntniss der in der Seide enthaltenen eiweissartigen Körper (a contribution to advance the knowledge on albuminoidal bodies in the silk). Hoppe-Seyler’s Zeitschr Physiol Chem.

[CR82] Xie R, Li M, Lu S, Sheng W, Xie Y (2007). Preparation of sericin film and its cytocompatibility. Key Eng Mater.

